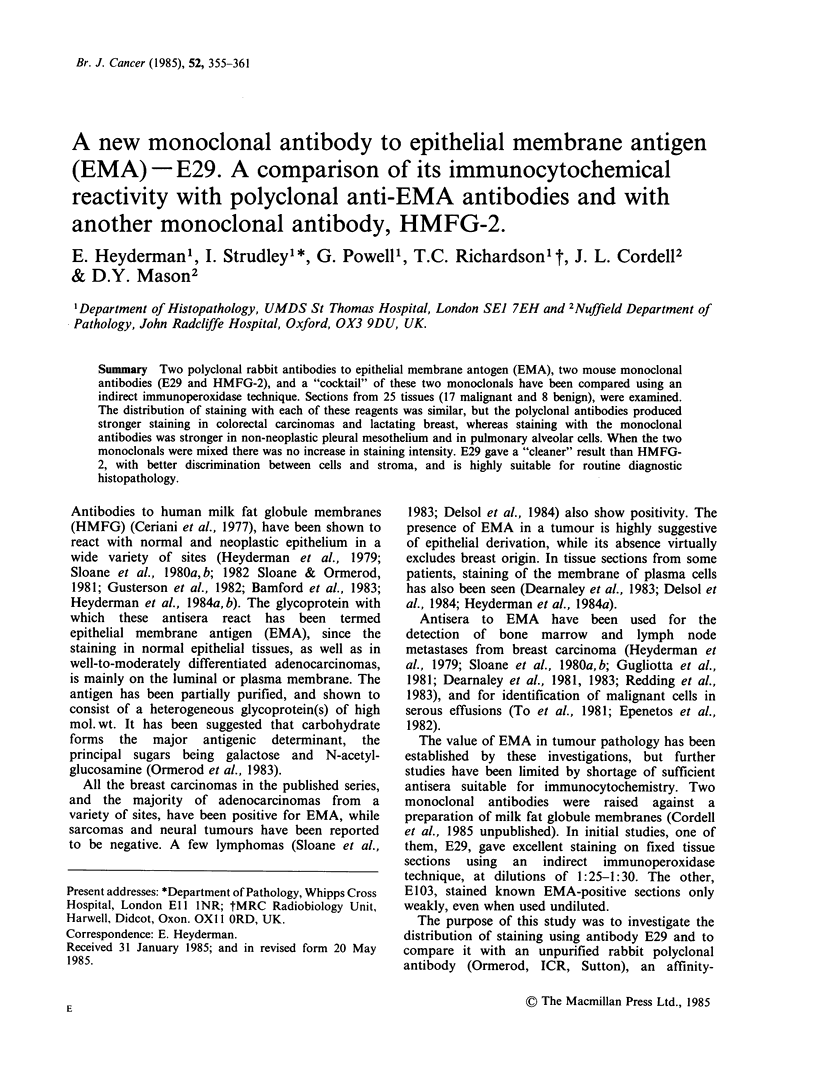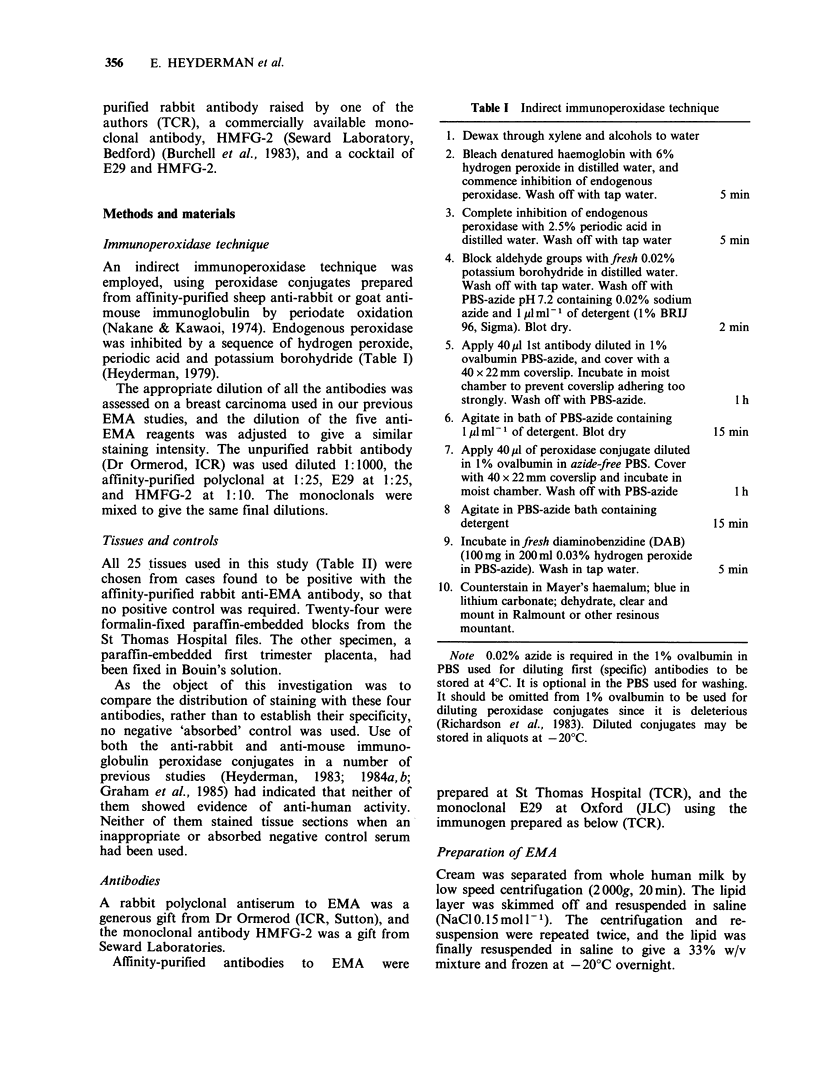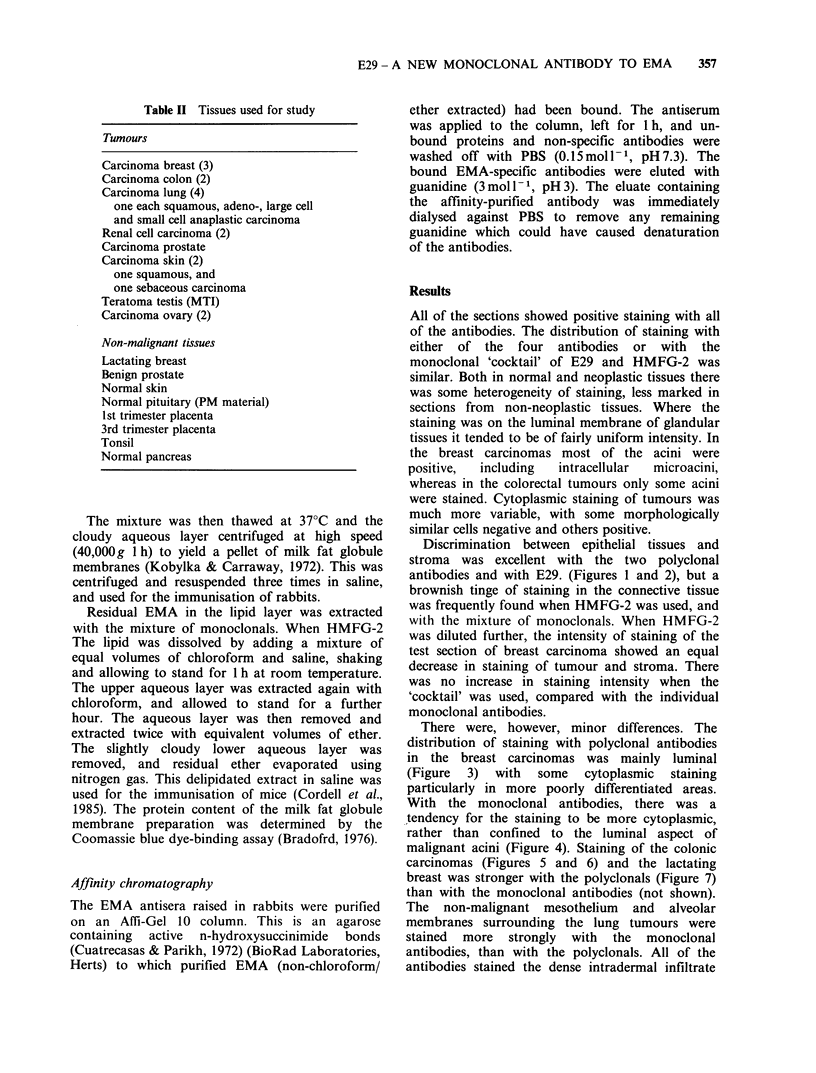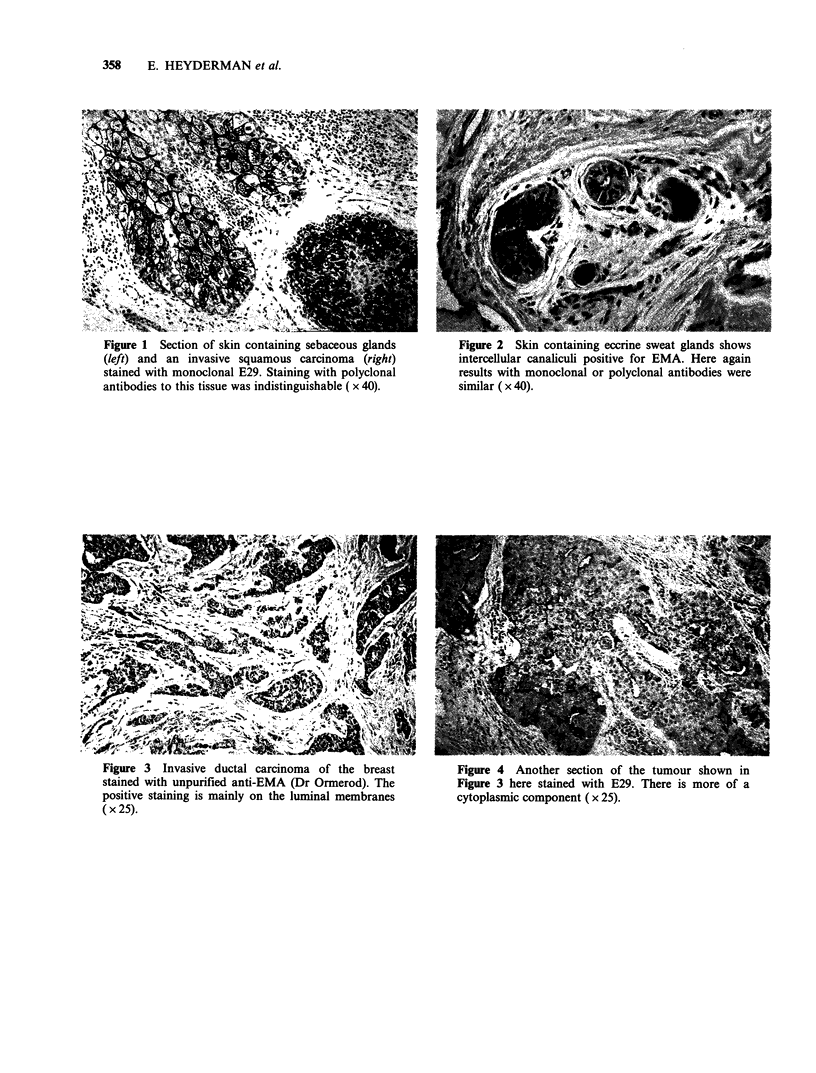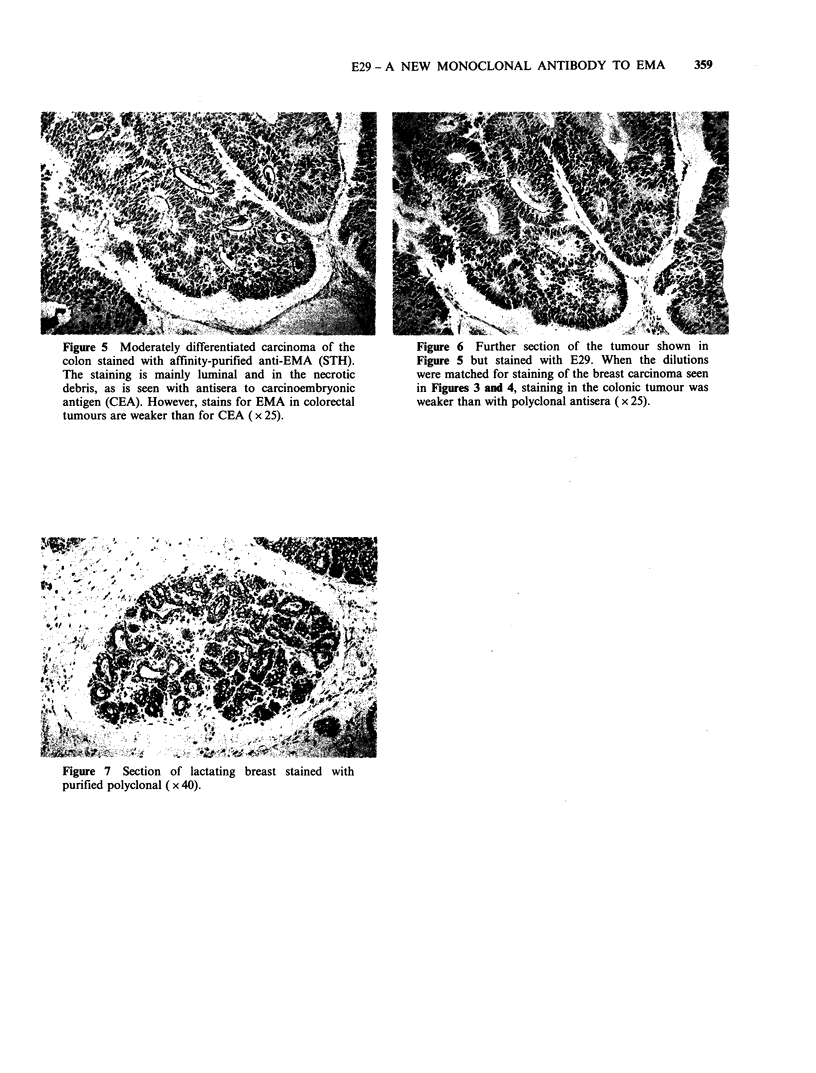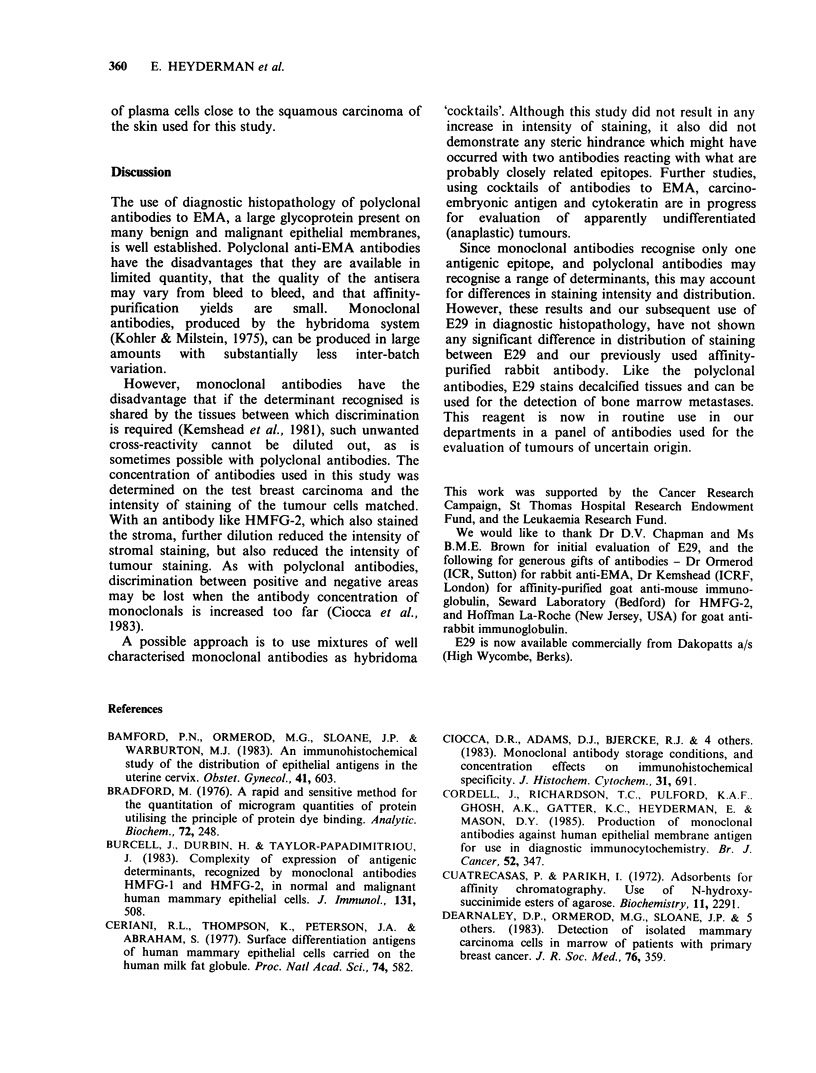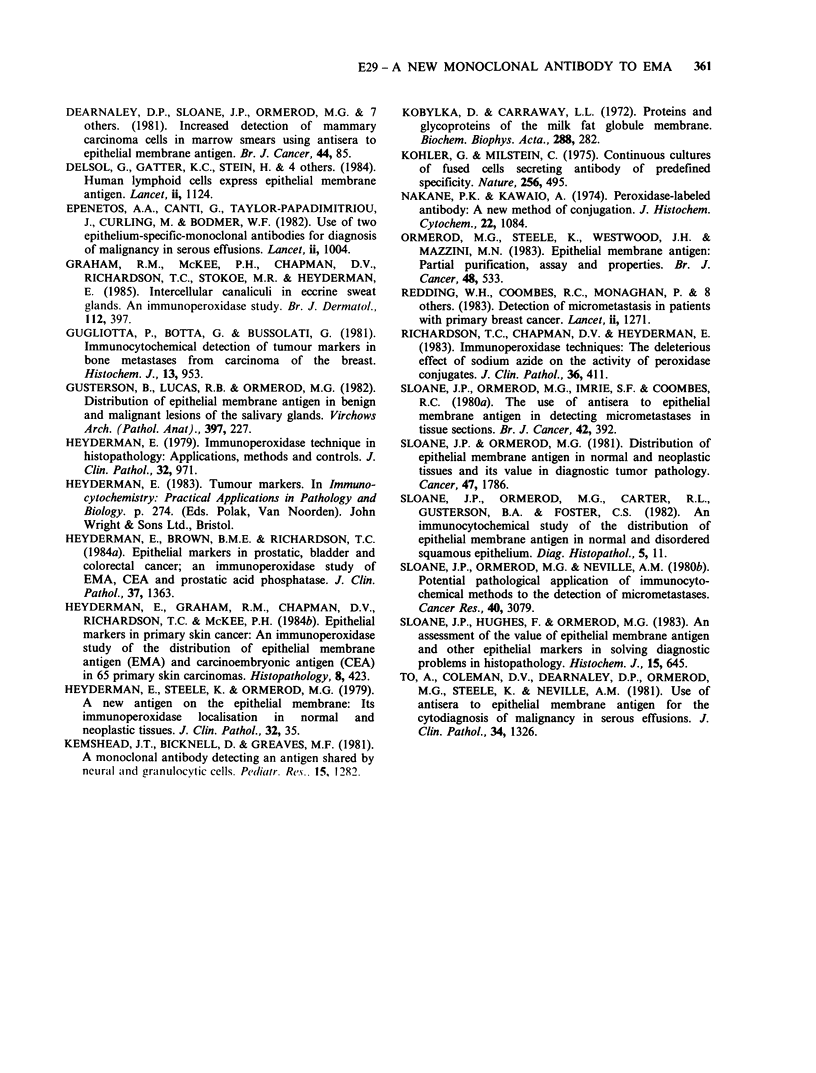# A new monoclonal antibody to epithelial membrane antigen (EMA)-E29. A comparison of its immunocytochemical reactivity with polyclonal anti-EMA antibodies and with another monoclonal antibody, HMFG-2.

**DOI:** 10.1038/bjc.1985.201

**Published:** 1985-09

**Authors:** E. Heyderman, I. Strudley, G. Powell, T. C. Richardson, J. L. Cordell, D. Y. Mason

## Abstract

**Images:**


*Br. J. Cancer* (1985), **52**, 355–361

### A new monoclonal antibody to epithelial membrane antigen (EMA) — E29. A comparison of its immunocytochemical reactivity with polyclonal anti-EMA antibodies and with another monoclonal antibody, HMFG-2.

E. Heyderman[1], I. Strudley[1]*, G. Powell[1], T.C. Richardson[1]†, J. L. Cordell[2] & D.Y. Mason[2]

[1]*Department of Histopathology, UMDS St Thomas Hospital, London SE1 7EH and* [2]*Nuffield Department of Pathology, John Radcliffe Hospital, Oxford, OX3 9DU, UK.*

**Summary** Two polyclonal rabbit antibodies to epithelial membrane antogen (EMA), two mouse monoclonal antibodies (E29 and HMFG-2), and a "cocktail" of these two monoclonals have been compared using an indirect immunoperoxidase technique. Sections from 25 tissues (17 malignant and 8 benign), were examined. The distribution of staining with each of these reagents was similar, but the polyclonal antibodies produced stronger staining in colorectal carcinomas and lactating breast, whereas staining with the monoclonal antibodies was stronger in non-neoplastic pleural mesothelium and in pulmonary alveolar cells. When the two monoclonals were mixed there was no increase in staining intensity. E29 gave a "cleaner" result than HMFG-2, with better discrimination between cells and stroma, and is highly suitable for routine diagnostic histopathology.

Antibodies to human milk fat globule membranes (HMFG) (Ceriani *et al.*, 1977), have been shown to react with normal and neoplastic epithelium in a wide variety of sites (Heyderman *et al.*, 1979; Sloane *et al.*, 1980*a,b*; 1982 Sloane & Ormerod, 1981; Gusterson *et al.*, 1982; Bamford *et al.*, 1983; Heyderman *et al.*, 1984*a,b*). The glycoprotein with which these antisera react has been termed epithelial membrane antigen (EMA), since the staining in normal epithelial tissues, as well as in well-to-moderately differentiated adenocarcinomas, is mainly on the luminal or plasma membrane. The antigen has been partially purified, and shown to consist of a heterogeneous glycoprotein(s) of high mol. wt. It has been suggested that carbohydrate forms the major antigenic determinant, the principal sugars being galactose and N-acetyl-glucosamine (Ormerod *et al.*, 1983).

All the breast carcinomas in the published series, and the majority of adenocarcinomas from a variety of sites, have been positive for EMA, while sarcomas and neural tumours have been reported to be negative. A few lymphomas (Sloane *et al.*, 1983; Delsol *et al.*, 1984) also show positivity. The presence of EMA in a tumour is highly suggestive of epithelial derivation, while its absence virtually excludes breast origin. In tissue sections from some patients, staining of the membrane of plasma cells has also been seen (Dearnaley *et al.*, 1983; Delsol *et al.*, 1984; Heyderman *et al.*, 1984*a*).

Antisera to EMA have been used for the detection of bone marrow and lymph node metastases from breast carcinoma (Heyderman *et al.*, 1979; Sloane *et al.*, 1980*a,b*; Gugliotta *et al.*, 1981; Dearnaley *et al.*, 1981, 1983; Redding *et al.*, 1983), and for identification of malignant cells in serous effusions (To *et al.*, 1981; Epenetos *et al.*, 1982).

The value of EMA in tumour pathology has been established by these investigations, but further studies have been limited by shortage of sufficient antisera suitable for immunocytochemistry. Two monoclonal antibodies were raised against a preparation of milk fat globule membranes (Cordell *et al.*, 1985 unpublished). In initial studies, one of them, E29, gave excellent staining on fixed tissue sections using an indirect immunoperoxidase technique, at dilutions of 1:25–1:30. The other, E103, stained known EMA-positive sections only weakly, even when used undiluted.

The purpose of this study was to investigate the distribution of staining using antibody E29 and to compare it with an unpurified rabbit polyclonal antibody (Ormerod, ICR, Sutton), an affinity-

Present addresses: *Department of Pathology, Whipps Cross Hospital, London E11 1NR; †MRC Radiobiology Unit, Harwell, Didcot, Oxon. OX11 0RD, UK.

Correspondence: E. Heyderman.

Received 31 January 1985; and in revised form 20 May 1985.

© The Macmillan Press Ltd., 1985

purified rabbit antibody raised by one of the authors (TCR), a commercially available monoclonal antibody, HMFG-2 (Seward Laboratory, Bedford) (Burchell *et al.*, 1983), and a cocktail of E29 and HMFG-2.

### Methods and materials

### *Immunoperoxidase technique*

An indirect immunoperoxidase technique was employed, using peroxidase conjugates prepared from affinity-purified sheep anti-rabbit or goat anti-mouse immunoglobulin by periodate oxidation (Nakane & Kawaoi, 1974). Endogenous peroxidase was inhibited by a sequence of hydrogen peroxide, periodic acid and potassium borohydride (Table I) (Heyderman, 1979).

The appropriate dilution of all the antibodies was assessed on a breast carcinoma used in our previous EMA studies, and the dilution of the five anti-EMA reagents was adjusted to give a similar staining intensity. The unpurified rabbit antibody (Dr Ormerod, ICR) was used diluted 1:1000, the affinity-purified polyclonal at 1:25, E29 at 1:25, and HMFG-2 at 1:10. The monoclonals were mixed to give the same final dilutions.

### *Tissues and controls*

All 25 tissues used in this study (Table II) were chosen from cases found to be positive with the affinity-purified rabbit anti-EMA antibody, so that no positive control was required. Twenty-four were formalin-fixed paraffin-embedded blocks from the St Thomas Hospital files. The other specimen, a paraffin-embedded first trimester placenta, had been fixed in Bouin's solution.

As the object of this investigation was to compare the distribution of staining with these four antibodies, rather than to establish their specificity, no negative 'absorbed' control was used. Use of both the anti-rabbit and anti-mouse immunoglobulin peroxidase conjugates in a number of previous studies (Heyderman, 1983; 1984*a*, *b*; Graham *et al.*, 1985) had indicated that neither of them showed evidence of anti-human activity. Neither of them stained tissue sections when an inappropriate or absorbed negative control serum had been used.

### *Antibodies*

A rabbit polyclonal antiserum to EMA was a generous gift from Dr Ormerod (ICR, Sutton), and the monoclonal antibody HMFG-2 was a gift from Seward Laboratories.

Affinity-purified antibodies to EMA were prepared at St Thomas Hospital (TCR), and the monoclonal E29 at Oxford (JLC) using the immunogen prepared as below (TCR).

**Table I** Indirect immunoperoxidase technique

| | | |
|---|---|---|
| 1. | Dewax through xylene and alcohols to water | |
| 2. | Bleach denatured haemoglobin with 6% hydrogen peroxide in distilled water, and commence inhibition of endogenous peroxidase. Wash off with tap water. | 5 min |
| 3. | Complete inhibition of endogenous peroxidase with 2.5% periodic acid in distilled water. Wash off with tap water | 5 min |
| 4. | Block aldehyde groups with *fresh* 0.02% potassium borohydride in distilled water. Wash off with tap water. Wash off with PBS-azide pH 7.2 containing 0.02% sodium azide and 1 $\mu$l ml$^{-1}$ of detergent (1% BRIJ 96, Sigma). Blot dry. | 2 min |
| 5. | Apply 40 $\mu$l 1st antibody diluted in 1% ovalbumin PBS-azide, and cover with a 40 x 22 mm coverslip. Incubate in moist chamber to prevent coverslip adhering too strongly. Wash off with PBS-azide. | 1 h |
| 6. | Agitate in bath of PBS-azide containing 1 $\mu$l ml$^{-1}$ of detergent. Blot dry | 15 min |
| 7. | Apply 40 $\mu$l of peroxidase conjugate diluted in 1% ovalbumin in *azide-free* PBS. Cover with 40 x 22 mm coverslip and incubate in moist chamber. Wash off with PBS-azide | 1 h |
| 8 | Agitate in PBS-azide bath containing detergent | 15 min |
| 9. | Incubate in *fresh* diaminobenzidine (DAB) (100 mg in 200 ml 0.03% hydrogen peroxide in PBS-azide). Wash in tap water. | 5 min |
| 10. | Counterstain in Mayer's haemalum; blue in lithium carbonate; dehydrate, clear and mount in Ralmount or other resinous mountant. | |

*Note* 0.02% azide is required in the 1% ovalbumin in PBS used for diluting first (specific) antibodies to be stored at 4°C. It is optional in the PBS used for washing. It should be omitted from 1% ovalbumin to be used for diluting peroxidase conjugates since it is deleterious (Richardson *et al.*, 1983). Diluted conjugates may be stored in aliquots at −20°C.

### *Preparation of EMA*

Cream was separated from whole human milk by low speed centrifugation (2 000*g*, 20 min). The lipid layer was skimmed off and resuspended in saline (NaCl 0.15 mol l$^{-1}$). The centrifugation and resuspension were repeated twice, and the lipid was finally resuspended in saline to give a 33% w/v mixture and frozen at −20°C overnight.

**Table II** Tissues used for study

| *Tumours* |
|---|
| Carcinoma breast (3) |
| Carcinoma colon (2) |
| Carcinoma lung (4) |
| one each squamous, adeno-, large cell and small cell anaplastic carcinoma |
| Renal cell carcinoma (2) |
| Carcinoma prostate |
| Carcinoma skin (2) |
| one squamous, and one sebaceous carcinoma |
| Teratoma testis (MTI) |
| Carcinoma ovary (2) |
| *Non-malignant tissues* |
| Lactating breast |
| Benign prostate |
| Normal skin |
| Normal pituitary (PM material) |
| 1st trimester placenta |
| 3rd trimester placenta |
| Tonsil |
| Normal pancreas |

The mixture was then thawed at 37°C and the cloudy aqueous layer centrifuged at high speed (40,000*g* 1 h) to yield a pellet of milk fat globule membranes (Kobylka & Carraway, 1972). This was centrifuged and resuspended three times in saline, and used for the immunisation of rabbits.

Residual EMA in the lipid layer was extracted with the mixture of monoclonals. When HMFG-2 The lipid was dissolved by adding a mixture of equal volumes of chloroform and saline, shaking and allowing to stand for 1 h at room temperature. The upper aqueous layer was extracted again with chloroform, and allowed to stand for a further hour. The aqueous layer was then removed and extracted twice with equivalent volumes of ether. The slightly cloudy lower aqueous layer was removed, and residual ether evaporated using nitrogen gas. This delipidated extract in saline was used for the immunisation of mice (Cordell *et al.*, 1985). The protein content of the milk fat globule membrane preparation was determined by the Coomassie blue dye-binding assay (Bradofrd, 1976).

### *Affinity chromatography*

The EMA antisera raised in rabbits were purified on an Affi-Gel 10 column. This is an agarose containing active n-hydroxysuccinimide bonds (Cuatrecasas & Parikh, 1972) (BioRad Laboratories, Herts) to which purified EMA (non-chloroform/ether extracted) had been bound. The antiserum was applied to the column, left for 1 h, and unbound proteins and non-specific antibodies were washed off with PBS ($0.15\,\mathrm{mol\,l^{-1}}$, pH 7.3). The bound EMA-specific antibodies were eluted with guanidine ($3\,\mathrm{mol\,l^{-1}}$, pH 3). The eluate containing the affinity-purified antibody was immediately dialysed against PBS to remove any remaining guanidine which could have caused denaturation of the antibodies.

### Results

All of the sections showed positive staining with all of the antibodies. The distribution of staining with either of the four antibodies or with the monoclonal 'cocktail' of E29 and HMFG-2 was similar. Both in normal and neoplastic tissues there was some heterogeneity of staining, less marked in sections from non-neoplastic tissues. Where the staining was on the luminal membrane of glandular tissues it tended to be of fairly uniform intensity. In the breast carcinomas most of the acini were positive, including intracellular microacini, whereas in the colorectal tumours only some acini were stained. Cytoplasmic staining of tumours was much more variable, with some morphologically similar cells negative and others positive.

Discrimination between epithelial tissues and stroma was excellent with the two polyclonal antibodies and with E29. (Figures 1 and 2), but a brownish tinge of staining in the connective tissue was frequently found when HMFG-2 was used, and with the mixture of monoclonals. When HMFG-2 was diluted further, the intensity of staining of the test section of breast carcinoma showed an equal decrease in staining of tumour and stroma. There was no increase in staining intensity when the 'cocktail' was used, compared with the individual monoclonal antibodies.

There were, however, minor differences. The distribution of staining with polyclonal antibodies in the breast carcinomas was mainly luminal (Figure 3) with some cytoplasmic staining particularly in more poorly differentiated areas. With the monoclonal antibodies, there was a tendency for the staining to be more cytoplasmic, rather than confined to the luminal aspect of malignant acini (Figure 4). Staining of the colonic carcinomas (Figures 5 and 6) and the lactating breast was stronger with the polyclonals (Figure 7) than with the monoclonal antibodies (not shown). The non-malignant mesothelium and alveolar membranes surrounding the lung tumours were stained more strongly with the monoclonal antibodies, than with the polyclonals. All of the antibodies stained the dense intradermal infiltrate

**Figure 1** Section of skin containing sebaceous glands (*left*) and an invasive squamous carcinoma (*right*) stained with monoclonal E29. Staining with polyclonal antibodies to this tissue was indistinguishable ( × 40).

**Figure 2** Skin containing eccrine sweat glands shows intercellular canaliculi positive for EMA. Here again results with monoclonal or polyclonal antibodies were similar ( × 40).

**Figure 3** Invasive ductal carcinoma of the breast stained with unpurified anti-EMA (Dr Ormerod). The positive staining is mainly on the luminal membranes ( × 25).

**Figure 4** Another section of the tumour shown in **Figure 3** here stained with E29. There is more of a cytoplasmic component ( × 25).

**Figure 5** Moderately differentiated carcinoma of the colon stained with affinity-purified anti-EMA (STH). The staining is mainly luminal and in the necrotic debris, as is seen with antisera to carcinoembryonic antigen (CEA). However, stains for EMA in colorectal tumours are weaker than for CEA ( × 25).

**Figure 6** Further section of the tumour shown in **Figure 5** but stained with E29. When the dilutions were matched for staining of the breast carcinoma seen in **Figures 3 and 4**, staining in the colonic tumour was weaker than with polyclonal antisera ( × 25).

**Figure 7** Section of lactating breast stained with purified polyclonal ( × 40).

of plasma cells close to the squamous carcinoma of the skin used for this study.

### Discussion

The use of diagnostic histopathology of polyclonal antibodies to EMA, a large glycoprotein present on many benign and malignant epithelial membranes, is well established. Polyclonal anti-EMA antibodies have the disadvantages that they are available in limited quantity, that the quality of the antisera may vary from bleed to bleed, and that affinity-purification yields are small. Monoclonal antibodies, produced by the hybridoma system (Kohler & Milstein, 1975), can be produced in large amounts with substantially less inter-batch variation.

However, monoclonal antibodies have the disadvantage that if the determinant recognised is shared by the tissues between which discrimination is required (Kemshead *et al.*, 1981), such unwanted cross-reactivity cannot be diluted out, as is sometimes possible with polyclonal antibodies. The concentration of antibodies used in this study was determined on the test breast carcinoma and the intensity of staining of the tumour cells matched. With an antibody like HMFG-2, which also stained the stroma, further dilution reduced the intensity of stromal staining, but also reduced the intensity of tumour staining. As with polyclonal antibodies, discrimination between positive and negative areas may be lost when the antibody concentration of monoclonals is increased too far (Ciocca *et al.*, 1983).

A possible approach is to use mixtures of well characterised monoclonal antibodies as hybridoma 'cocktails'. Although this study did not result in any increase in intensity of staining, it also did not demonstrate any steric hindrance which might have occurred with two antibodies reacting with what are probably closely related epitopes. Further studies, using cocktails of antibodies to EMA, carcino-embryonic antigen and cytokeratin are in progress for evaluation of apparently undifferentiated (anaplastic) tumours.

Since monoclonal antibodies recognise only one antigenic epitope, and polyclonal antibodies may recognise a range of determinants, this may account for differences in staining intensity and distribution. However, these results and our subsequent use of E29 in diagnostic histopathology, have not shown any significant difference in distribution of staining between E29 and our previously used affinity-purified rabbit antibody. Like the polyclonal antibodies, E29 stains decalcified tissues and can be used for the detection of bone marrow metastases. This reagent is now in routine use in our departments in a panel of antibodies used for the evaluation of tumours of uncertain origin.

This work was supported by the Cancer Research Campaign, St Thomas Hospital Research Endowment Fund, and the Leukaemia Research Fund.

We would like to thank Dr D.V. Chapman and Ms B.M.E. Brown for initial evaluation of E29, and the following for generous gifts of antibodies – Dr Ormerod (ICR, Sutton) for rabbit anti-EMA, Dr Kemshead (ICRF, London) for affinity-purified goat anti-mouse immunoglobulin, Seward Laboratory (Bedford) for HMFG-2, and Hoffman La-Roche (New Jersey, USA) for goat anti-rabbit immunoglobulin.

E29 is now available commercially from Dakopatts a/s (High Wycombe, Berks).

### References

BAMFORD, P.N., ORMEROD, M.G., SLOANE, J.P. & WARBURTON, M.J. (1983). An immunohistochemical study of the distribution of epithelial antigens in the uterine cervix. *Obstet. Gynecol.*, **41**, 603.

BRADFORD, M. (1976). A rapid and sensitive method for the quantitation of microgram quantities of protein utilising the principle of protein dye binding. *Analytic. Biochem.*, **72**, 248.

BURCELL, J., DURBIN, H. & TAYLOR-PAPADIMITRIOU, J. (1983). Complexity of expression of antigenic determinants, recognized by monoclonal antibodies HMFG-1 and HMFG-2, in normal and malignant human mammary epithelial cells. *J. Immunol.*, **131**, 508.

CERIANI, R.L., THOMPSON, K., PETERSON, J.A. & ABRAHAM, S. (1977). Surface differentiation antigens of human mammary epithelial cells carried on the human milk fat globule. *Proc. Natl Acad. Sci.*, **74**, 582.

CIOCCA, D.R., ADAMS, D.J., BJERCKE, R.J. & 4 others. (1983). Monoclonal antibody storage conditions, and concentration effects on immunohistochemical specificity. *J. Histochem. Cytochem.*, **31**, 691.

CORDELL, J., RICHARDSON, T.C., PULFORD, K.A.F., GHOSH, A.K., GATTER, K.C., HEYDERMAN, E. & MASON, D.Y. (1985). Production of monoclonal antibodies against human epithelial membrane antigen for use in diagnostic immunocytochemistry. *Br. J. Cancer*, **52**, 347.

CUATRECASAS, P. & PARIKH, I. (1972). Adsorbents for affinity chromatography. Use of N-hydroxy-succinimide esters of agarose. *Biochemistry*, **11**, 2291.

DEARNALEY, D.P., ORMEROD, M.G., SLOANE, J.P. & 5 others. (1983). Detection of isolated mammary carcinoma cells in marrow of patients with primary breast cancer. *J. R. Soc. Med.*, **76**, 359.

DEARNALEY, D.P., SLOANE, J.P., ORMEROD, M.G. & 7 others. (1981). Increased detection of mammary carcinoma cells in marrow smears using antisera to epithelial membrane antigen. *Br. J. Cancer*, **44**, 85.

DELSOL, G., GATTER, K.C., STEIN, H. & 4 others. (1984). Human lymphoid cells express epithelial membrane antigen. *Lancet*, **ii**, 1124.

EPENETOS, A.A., CANTI, G., TAYLOR-PAPADIMITRIOU, J., CURLING, M. & BODMER, W.F. (1982). Use of two epithelium-specific-monoclonal antibodies for diagnosis of malignancy in serous effusions. *Lancet*, **ii**, 1004.

GRAHAM, R.M., McKEE, P.H., CHAPMAN, D.V., RICHARDSON, T.C., STOKOE, M.R. & HEYDERMAN, E. (1985). Intercellular canaliculi in eccrine sweat glands. An immunoperoxidase study. *Br. J. Dermatol.*, **112**, 397.

GUGLIOTTA, P., BOTTA, G. & BUSSOLATI, G. (1981). Immunocytochemical detection of tumour markers in bone metastases from carcinoma of the breast. *Histochem. J.*, **13**, 953.

GUSTERSON, B., LUCAS, R.B. & ORMEROD, M.G. (1982). Distribution of epithelial membrane antigen in benign and malignant lesions of the salivary glands. *Virchows Arch. (Pathol. Anat).*, **397**, 227.

HEYDERMAN, E. (1979). Immunoperoxidase technique in histopathology: Applications, methods and controls. *J. Clin. Pathol.*, **32**, 971.

HEYDERMAN, E. (1983). Tumour markers. In *Immunocytochemistry: Practical Applications in Pathology and Biology*. p. 274. (Eds. Polak, Van Noorden). John Wright & Sons Ltd., Bristol.

HEYDERMAN, E., BROWN, B.M.E. & RICHARDSON, T.C. (1984*a*). Epithelial markers in prostatic, bladder and colorectal cancer; an immunoperoxidase study of EMA, CEA and prostatic acid phosphatase. *J. Clin. Pathol.*, **37**, 1363.

HEYDERMAN, E., GRAHAM, R.M., CHAPMAN, D.V., RICHARDSON, T.C. & McKEE, P.H. (1984*b*). Epithelial markers in primary skin cancer: An immunoperoxidase study of the distribution of epithelial membrane antigen (EMA) and carcinoembryonic antigen (CEA) in 65 primary skin carcinomas. *Histopathology*, **8**, 423.

HEYDERMAN, E., STEELE, K. & ORMEROD, M.G. (1979). A new antigen on the epithelial membrane: Its immunoperoxidase localisation in normal and neoplastic tissues. *J. Clin. Pathol.*, **32**, 35.

KEMSHEAD, J.T., BICKNELL, D. & GREAVES, M.F. (1981). A monoclonal antibody detecting an antigen shared by neural and granulocytic cells. *Pediatr. Res.*, **15**, 1282.

KOBYLKA, D. & CARRAWAY, L.L. (1972). Proteins and glycoproteins of the milk fat globule membrane. *Biochem. Biophys. Acta.*, **288**, 282.

KOHLER, G. & MILSTEIN, C. (1975). Continuous cultures of fused cells secreting antibody of predefined specificity. *Nature*, **256**, 495.

NAKANE, P.K. & KAWAIO, A. (1974). Peroxidase-labeled antibody: A new method of conjugation. *J. Histochem. Cytochem.*, **22**, 1084.

ORMEROD, M.G., STEELE, K., WESTWOOD, J.H. & MAZZINI, M.N. (1983). Epithelial membrane antigen: Partial purification, assay and properties. *Br. J. Cancer*, **48**, 533.

REDDING, W.H., COOMBES, R.C., MONAGHAN, P. & 8 others. (1983). Detection of micrometastasis in patients with primary breast cancer. *Lancet*, **ii**, 1271.

RICHARDSON, T.C., CHAPMAN, D.V. & HEYDERMAN, E. (1983). Immunoperoxidase techniques: The deleterious effect of sodium azide on the activity of peroxidase conjugates. *J. Clin. Pathol.*, **36**, 411.

SLOANE, J.P., ORMEROD, M.G., IMRIE, S.F. & COOMBES, R.C. (1980*a*). The use of antisera to epithelial membrane antigen in detecting micrometastases in tissue sections. *Br. J. Cancer*, **42**, 392.

SLOANE, J.P. & ORMEROD, M.G. (1981). Distribution of epithelial membrane antigen in normal and neoplastic tissues and its value in diagnostic tumor pathology. *Cancer*, **47**, 1786.

SLOANE, J.P., ORMEROD, M.G., CARTER, R.L., GUSTERSON, B.A. & FOSTER, C.S. (1982). An immunocytochemical study of the distribution of epithelial membrane antigen in normal and disordered squamous epithelium. *Diag. Histopathol.*, **5**, 11.

SLOANE, J.P., ORMEROD, M.G. & NEVILLE, A.M. (1980*b*). Potential pathological application of immunocytochemical methods to the detection of micrometastases. *Cancer Res.*, **40**, 3079.

SLOANE, J.P., HUGHES, F. & ORMEROD, M.G. (1983). An assessment of the value of epithelial membrane antigen and other epithelial markers in solving diagnostic problems in histopathology. *Histochem. J.*, **15**, 645.

TO, A., COLEMAN, D.V., DEARNALEY, D.P., ORMEROD, M.G., STEELE, K. & NEVILLE, A.M. (1981). Use of antisera to epithelial membrane antigen for the cytodiagnosis of malignancy in serous effusions. *J. Clin. Pathol.*, **34**, 1326.